# Functional confirmation that the R1488* variant in *SCN9A* results in complete loss-of-function of Na_v_1.7

**DOI:** 10.1186/s12881-018-0643-4

**Published:** 2018-07-23

**Authors:** Wen He, Gareth T. Young, Baohong Zhang, Peter J. Cox, Lily Ting-Yin Cho, Sally John, Sara A. Paciga, Linda S. Wood, Nicolas Danziger, Serena Scollen, Ciara Vangjeli

**Affiliations:** 1 Worldwide Research & Development, Pfizer Inc, Eastern Point Road, Groton, CT 06340 USA; 2 Pfizer Ltd, The Portway Building, Granta Park, Great Abington, Cambridge, CB21 6GS UK; 3 Pfizer Inc, 300 Technology Square, Cambridge, MA 02139 USA; 4 Pain Center, Groupe Hospitalier Pitié-Salpêtrière, Paris, France

**Keywords:** Pain, *SCN9A*, Na_v_1.7, SNV, Whole exome sequencing

## Abstract

**Background:**

Individuals with an extremely rare inherited condition, termed Congenital Insensitivity to Pain (CIP), do not feel pain in response to noxious stimuli. Variants in *SCN9A*, encoding the transmembrane voltage-gated sodium channel Na_v_1.7, have previously been reported in subjects with CIP accompanied by anosmia, which are typically transmitted in a recessive pattern. Functional characterisations of some of these *SCN9A* mutations show that they result in complete loss-of-function of Na_v_1.7.

**Methods:**

In a consanguineous family we performed whole exome sequencing of three members who have a diagnosis of CIP and one unaffected family member. The functional effects of the segregating variant in *SCN9A* were determined using patch clamp electrophysiology in human embryonic kidney (HEK) 293 cells transfected with the variant.

**Results:**

We found that each CIP subject was homozygous for a putatively nonsense variant, R1488*, in *SCN9A*. This variant was reported elsewhere in a subject with CIP, though the functional effect was not determined. Using electrophysiology, we confirm that this variant results in a complete loss-of-function of Na_v_1.7.

**Conclusions:**

We confirm through electrophysiological analysis that this R1488* variant in *SCN9A* results in complete loss-of-function of Na_v_1.7, which is consistent with reports on other variants in this gene in subjects with CIP.

## Background

All mammals need to detect and avoid noxious stimuli to protect their tissue from damage; this is achieved via nociception. To date, over 30 loss-of-function variants in *SCN9A*, encoding the Na_v_1.7 sodium channel, have been identified and reported to cause Congenital Insensitivity to Pain (CIP), a very rare autosomal recessive disorder characterised by the complete absence of pain perception [1, 2]. Conversely, gain-of-function variants in *SCN9A* have been reported in patients with rare extreme pain disorders such as inherited erythromyelgia and paroxysmal episodic pain disorder [3].

CIP is present from birth with painless injuries evident from early in infancy such as damage to the tongue and lips caused by biting. Patients are often anosmic but otherwise have normal sensory modalities. Perception of passive movement, joint position, and vibration are normal, as are tactile thresholds and light touch perception. Reflexes and autonomic responses are also normal. The disorder is considered to be an irreversible disorder of peripheral pain sensing neurons; why these neurons are so reliant on Na_v_1.7, however, is not clear.

We describe a consanguineous three-generation pedigree in which CIP was observed in three individuals over two generations. The primary hypothesis was that the causative variant would be homozygous in the affected family members, given the consanguinity and the recessive pattern of inheritance observed. Exome sequencing was employed to identify variants that segregate accordingly. We identified a homozygous nonsense variant R1488* in *SCN9A* in all three affected subjects and showed that this variant results in complete loss-of-function of the Na_v_1.7 ion channel.

## Methods

### Families and sampling

**Fig. 1:**
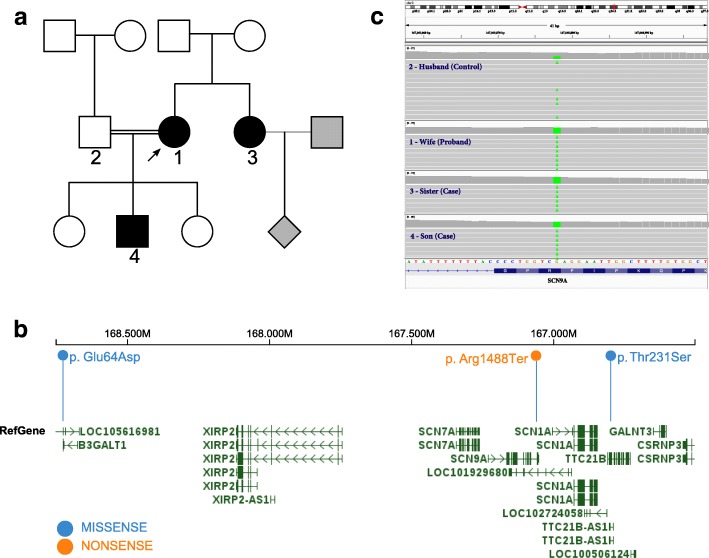
Pedigree and identification of the variant. **a** Pedigree of the family showing the relationship between affected and unaffected members. Grey line denotes unknown relationship (level of consanguinity), grey shading denotes unknown phenotype. **b** Chromosomal location of three variants identified within 1.9 Mb on chromosome 2. The orange text denotes the *SCN9A* nonsense variant. Blue text denotes the two other rare homozygous missense variants identified in the region. **c** Integrative Genomics Viewer (IGV) visualization of the *SCN9A* nonsense variant in four tested subjects

**Fig. 2:**
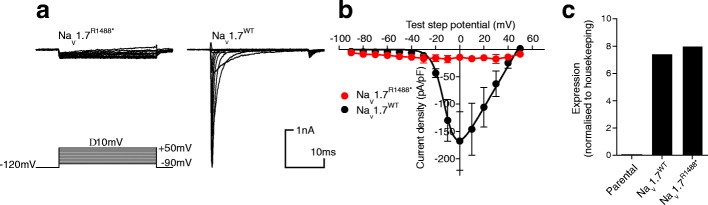
Na_v_1.7^R1488*^ is a non-functional ion channel. **a** Representative current traces in response to activating voltage steps (lower panel). No inward currents were observed in any cells expressing Na_v_1.7^R1488*^ (left panel, 0/20 cells) whereas prominent inactivating currents were observed in cells expressing Na_v_1.7^WT^ (right panel, 16/20 cells). **b** Current-voltage relationship for cells expressing Na_v_1.7^R1488*^ show no detectable current for the mutant whereas cells expressing Na_v_1.7^WT^ exhibited a clear current-voltage relationship typical for voltage-gated sodium channels (− 13.0 ± 0.8 mV, *n* = 10). **c** Quantitative PCR shows that both Na_v_1.7^R1488*^ and Na_v_1.7^WT^ cells express comparable amount of mRNA demonstrating that the variant is likely to be non-functional at the protein level. No mRNA is detectable in a parental cell line

The index case (Fig. 1a, 1) was described previously [4]. She presented with a history of pain free wounds, burns, bone fractures, and appendicitis and experienced no pain during childbirth. In addition, this individual had other neurological dysfunctions: anosmia, aguesia, unexplained hyperthermia and impaired cold and warm perception. The painless phenotype was inherited recessively whereby her only son was affected but two daughters were unaffected. The index case and her husband (Fig. 1a, 2) were first cousins - he is the son of her mother’s brother. The son (Fig. 1a, 4) had a history of painless burns, wounds, self-mutilation, fractures and infections. He was also anosmic and was reported to be hyperactive. The third affected family member was a sister (Fig. 1a, 3) of the index case. She experienced similar painless, noxious events characteristic of CIP: wounds, burns, self-mutilation of the lips and was also anosmic. None of the affected subjects showed any cognitive or motor dysfunction. The two affected females reported residual pain experiences of differing severities. The index case reported one episode of a tension-type headache. Her sister (Fig. 1a, 3) reported bowel pain and some pain perception since adolescence and following pregnancy, she reported being hypersensitive to pain stimuli and to odourants. The son had no reports of residual pain but was in his first decade of life at the time of reporting.

The husband (Figs. 1a and 2) and all other relatives shown in the pedigree reported that they experience pain in a ‘typical’ manner. DNA was available from the husband but not from any of the other unaffected family members.

Genomic DNA was isolated from peripheral blood by standard methods.

### Exome capture and analysis

Genomic DNA was prepared for whole exome sequencing using Agilent SureSelect version 4, 51 megabase (Mb) capture kit following the manufacturer’s protocol (Agilent Technologies). These samples were then sequenced using the Illumina HiSeq 2000 platform (Illumina, Inc., San Diego, CA; paired-end: 2x100bp) by Perkin Elmer Next Generation Sequencing and Analysis Center (Branford, CT).

The reads generated were mapped to NCBI Human Reference (GRCh37; hg19) using Burrows-Wheeler Aligner (BWA) v0.5.9 [5]. The alignment files for each sample were sorted and indexed by SAMtools [6] to create the final Binary Alignment Map (BAM) files, which is a compressed binary version of a Sequence Alignment Map (SAM) file. Variant discovery tool for high-throughput sequencing data Genome Analysis Toolkit (GATK) v1.0.5777 [7] was run using default values. Variant functional annotation tool ANNOVAR [8] was adopted for genomic annotation. To predict the effects of coding non-synonymous variants on protein function, we used both Sorting Intolerant From Tolerant (SIFT) [9] and Polymorphism Phenotyping version 2 (Polyphen-2) [10] in our search for the potential causal variants.

Minor allele frequency information was downloaded from online databases of genetic variants: NCBI dbSNP, 1000 genomes [11], and NHLBI Exome Sequencing Project (ESP) Exome Variant Server [12] and were used in the variant filtering process. The upper minor allele frequency cut-off was 1%. Individual variants were evaluated in more detail by visualization of the BAM files with the Integrative Genomics Viewer (IGV) [13].

### Molecular biology and cell line generation

The human Na_v_1.7 expression construct in the pLNCX2 retroviral vector (Clontech) has been described previously [14]. Polymerase Chain Reaction (PCR)-based site-directed mutagenesis was performed to introduce a stop codon at R1488 of the Na_v_1.7 protein. Stable cell lines expressing Na_v_1.7^WT^ and Na_v_1.7^R1488*^ were produced as follows. Briefly, retroviruses were generated from sequence verified wild type and mutant constructs by transfecting the constructs into a GP2 packaging cell line (Clontech) using Lipofectamine 2000 reagent (Invitrogen). Retroviruses were harvested 48 h later and purified by centrifugation at 1500 g to remove residual GP2 cells. Native voltage-gated sodium channels comprise of large, channel forming, 24 transmembrane domain α-subunits. In a neuronal context, these α-subunits form co-complex with β-subunits, which aid the expression and trafficking of the α-subunit to the plasma membrane. To reproduce this cellular process, β1 and β2 subunits were co-expressed. The viruses were transduced into HEK-293 cells co-expressing a chimera of Na_v_β1 and Na_v_β2 auxiliary subunits. Stable cell populations were generated by G418 (800 μg/ml) selection. The control is the wild type *SCN9A* gene which was stably expressed in HEK-293 cells using the same procedures.

### Patch-clamp electrophysiology

All recordings were performed at room temperature in HEK-293 cells expressing human Na_V_1.7. Patch-clamp recordings were made using a Multiclamp 700A amplifier and digitised by a Digidata 2000. Glass borosilicate pipettes were used (typical R_pip_ of 2–5 MΩ resulting in uncompensated R_series_ < 8 MΩ). Cells were recorded in “reduced sodium” extracellular solution (avoiding voltage errors resulting from large currents) containing (in mM) NaCl (30), ChoCl (80) KCl (4), CaCl2 (1.8), MgCl2 (1), HEPES (10), glucose (5); pH was adjusted to 7.4 with NaOH. Pipettes were filled with intracellular solutions (in mM) KCl (140), MgCl2 (1.6), MgATP (2.5), NaGTP (0.5), EGTA (2), HEPES (10); pH was adjusted to 7.3. An activation protocol was used whereby cells were voltage-clamped at a holding potential of −120 mV followed by a 50 ms step to the activating step (−90 mV to + 40 mV, 10 mV steps). The peak inward current at each activating step was quantified and presented as current-voltage curves.

### qPCR

Expression of *SCN9A* messenger RNA was assessed in the stable cell line used in this study as previously reported [15]. Taqman qPCR primers were used for *SCN9A* (Hs00161567) and for the housekeeping gene GAPDH (Hs02758991_g1). *SCN9A* expression was expressed as a ratio of GAPDH. A HEK-293 parental cell line not transfected with *SCN9A* was used as a negative control.

## Results

### Identification of a segregating *SCN9A* variant

After mapping all the reads, we identified that 94% of the targeted region of 51 million bases is covered at least once. Eighty-two percent of targeted region is covered for 20× or more. Each of the four individuals had >8000 putatively functional (nonsynonymous, nonsense or frameshift) variants. To filter out the non-causal variants, we first removed the homozygous variants found in the unaffected subject, followed by the homozygous variants not shared by all three affected subjects. The same filtering process was applied to heterozygous variants. As a result, we obtained 303 variants that were shared by all three affected subjects, but not by unaffected subject. Of these 303 variants, 11 variants have a minor allele frequency <1%, and three of these were homozygous in all three affected subjects and heterozygous in the unaffected subject. All three variants are co-located within 1.9 Mb on chromosome 2 (Fig. 1b). One of the three variants, an *SCN9A* nonsense variant p.(Arg1488*) or p.(R1488*) was found to be homozygous in all affected subjects and was in heterozygous form in the unaffected subject (Fig. 1c). The details of this variant, according to Human Genome Variation Society (HGVS) nomenclature [16] is NC_000002.11:g.167060878G > A NM_002977.2:c.4462C > T p.(Arg1488*). This variant is recorded in ClinVar with ID as 245799; and in dbSNP as rs187558439. This variant has been previously reported in a patient with CIP [2], but the variant was not functionally characterised. The other two rare homozygous variants identified are *TTC21B* nonsynonymous variant Thr231Ser (Clinvar ID: 198257, dbSNP ID: rs149925563); and *B3GALT1* nonsynonymous variant Glu64Asp (dbSNP ID: rs141683896). Neither gene has a known role in pain perception, thus we prioritised the exploration of the *SCN9A* variant.

### Na_v_1.7^R1488*^ is a non-functional ion channel

Stable cell lines were generated expressing the wild type (Na_v_1.7^WT^) or the variant (Na_v_1.7^R1488*^), and both were co-transfected with Na_v_β1 and Na_v_β2 to ensure membrane trafficking of the mature channel. Cell lines expressing Na_v_1.7^WT^ and Na_v_1.7^R1488*^ were assessed by whole-cell patch-clamp electrophysiology using a voltage-activation protocol (Fig. 2a). Cells expressing Na_v_1.7^WT^ exhibited large inward currents in response to depolarising voltages more positive than −40 mV with a V_1/2_ of activation of − 13.0 ± 0.8 mV (*n* = 10, Fig. 2b). Conversely, the same voltage protocols elicited no inward currents in Na_v_1.7^R1488*^ cells. When measuring inward currents at 0 mV there were no detectable currents in Na_v_1.7^R1488*^ cells (0/20 cells, >100 pA); whereas there were clear inward currents in Na_v_1.7^WT^ cells (16/20 cells, >100pA). To ensure both cell lines were expressing *SCN9A* mRNA at comparable levels we assessed expression using qPCR; both cell lines expressed identical levels of mRNA demonstrating that the lack of functional responses are not due to mRNA expression levels (Fig. 2c). The parental line in Fig. 2c is the non-transfected HEK-293 cell line.

Taken together, these data demonstrate that the truncation of Na_v_1.7 at R1488 results in a completely non-functional channel. Using the “50 nucleotide” rule which forms the basis of nonsense mediated decay (NMD) classifier [17], the R1488* variant is predicted to result in nonsense mediated decay. It is located at position 4809 of 9768 nucleotides in the transcript within exon 25 out of 27, and so satisfies the criterion of being greater than 50 nucleotides from the last exon junction. Overall, our findings are consistent with previous reports of the role of Na_v_1.7 ion channels in pain sensation.

## Discussion

Here we identify a *SCN9A* CIP variant present in the homozygous state in three individuals with CIP and demonstrate for the first time that this variant results in complete loss-of-function of the ion channel Na_v_1.7 encoded for by this gene. Electrophysiological analysis of the resulting Na_v_1.7^R1488*^ ion channel yielded no functional responses in comparison to Na_v_1.7^WT^, demonstrating that the CIP subjects had deficient Na_v_1.7 channels. This finding supports the body of human and mouse [18] evidence that knocking out *SCN9A* results in loss of pain perception. According to the annotation of Na_v_1.7 in UniProt (http://www.uniprot.org), R1488 is located within the cytoplasmic topological domain between repeats III and IV. Despite the qPCR results showing that mRNA expression is at a comparable level for both the variant and the wild type, without CIP subject-derived cells or genome editing of a human cell line with this point mutation we cannot rule out that the loss-of-function effect that we have observed is driven by NMD as would be predicted. It would be interesting to determine whether homozygous carriers of this variant had very low or completely absent expression of the Na_v_1.7 protein. Alternatively, these subjects may produce a truncated though inactive version of Na_v_1.7.

We adopted a hypothesis-free approach and identified three putative causative rare homozygous variants. Variants in *BGALT1* and *TTC21B* in addition to *SCN9A* were identified. However, the weight of evidence implicating *SCN9A* as a driver of CIP, along with the previous identification of this particular variant in a patient with CIP, and the functional evidence we present, strengthen the likelihood that CIP in this particular family is *SCN9A*-driven.

*SCN9A*-driven CIP is typically considered to be an immutable phenomenon, with patients suffering from lifelong painlessness accompanied by a lack of ability to smell. Intriguingly, the clinical features of the CIP patients do not entirely match with previously reported CIP caused by variants in *SCN9A*. The two affected females reported some residual pain experiences along with classical *SCN9A*-driven anosmia. One of the female subjects reported becoming hypersensitive to some painful stimuli following pregnancy and also regained hypersensitivity to odourants. A further example of an *SCN9A*-CIP patient experiencing pain is that of a female individual who began to experience neuropathic pain symptoms following pelvic fractures and an epidural haematoma [19]. We note that in this particular report the hip fracture was suffered as a consequence of bone weakening during pregnancy. So, similar to the case reported here, it appears that during pregnancy, dynamic changes can occur in the nervous system that compensate for a lack of *SCN9A* and return nociception and smell to the CIP individual.

A recent report has proposed that CIP associated with loss of Na_v_1.7 causes analgesia via an increase in expression of the enkephalin precursor gene *PENK*. CIP in humans was shown to be substantially reversed by the opioid antagonist naloxone supporting this hypothesis [20]. It is possible that *PENK*, or indeed other regulatory genes of the opioid system, are altered during pregnancy and so may be part of the underlying mechanism of our observations. However this does not explain why the index case, and other reported CIP female patients, did not have the same experience and so further work is required to test this hypothesis.

## Conclusion

This work demonstrates that the R1488* variant seen in patients with CIP results in complete loss-of-function of Na_v_1.7, providing strong evidence that this variant causes the CIP phenotype. In addition, this finding provides further confidence-in-rationale for the therapeutic strategy of blocking Na_v_1.7 for the treatment of pain.
